# Driver Take-Over Behaviour Study Based on Gaze Focalization and Vehicle Data in CARLA Simulator

**DOI:** 10.3390/s22249993

**Published:** 2022-12-19

**Authors:** Javier Araluce, Luis M. Bergasa, Manuel Ocaña, Elena López-Guillén, Rodrigo Gutiérrez-Moreno, J. Felipe Arango

**Affiliations:** Electronics Department, University of Alcalá, 28805 Alcalá de Henares, Spain

**Keywords:** gaze focalization, driving behaviour study, CARLA simulator, non-driving-related tasks (NDRTs), take-over time, take-over quality, driver situation awareness

## Abstract

Autonomous vehicles are the near future of the automobile industry. However, until they reach Level 5, humans and cars will share this intermediate future. Therefore, studying the transition between autonomous and manual modes is a fascinating topic. Automated vehicles may still need to occasionally hand the control to drivers due to technology limitations and legal requirements. This paper presents a study of driver behaviour in the transition between autonomous and manual modes using a CARLA simulator. To our knowledge, this is the first take-over study with transitions conducted on this simulator. For this purpose, we obtain driver gaze focalization and fuse it with the road’s semantic segmentation to track to where and when the user is paying attention, besides the actuators’ reaction-time measurements provided in the literature. To track gaze focalization in a non-intrusive and inexpensive way, we use a method based on a camera developed in previous works. We devised it with the OpenFace 2.0 toolkit and a NARMAX calibration method. It transforms the face parameters extracted by the toolkit into the point where the user is looking on the simulator scene. The study was carried out by different users using our simulator, which is composed of three screens, a steering wheel and pedals. We distributed this proposal in two different computer systems due to the computational cost of the simulator based on the CARLA simulator. The robot operating system (ROS) framework is in charge of the communication of both systems to provide portability and flexibility to the proposal. Results of the transition analysis are provided using state-of-the-art metrics and a novel driver situation-awareness metric for 20 users in two different scenarios.

## 1. Introduction

In recent years, the academy and industry have made significant improvements in the autonomous-driving paradigm. According to SAE (J3016), five levels of automation are applied, achieving full automation at Level 5. Nowadays, we have technologies that use functions traditionally reserved for the driver to take over. In Levels 1 and 2, the driver has to supervise the vehicle. In Levels 3 and 4, the driver does not need to have control at restricted moments or for most of the trip. Problems arise at above levels where the driver is in the loop and is not always aware of what is happening (Levels 1 and 2), or he is out of the loop and needs to quickly resume control (Levels 3 and 4) due to technology limitations or legal requirements (e.g., when the vehicle approaches a police checkpoint) [1].

**Figure 1:**
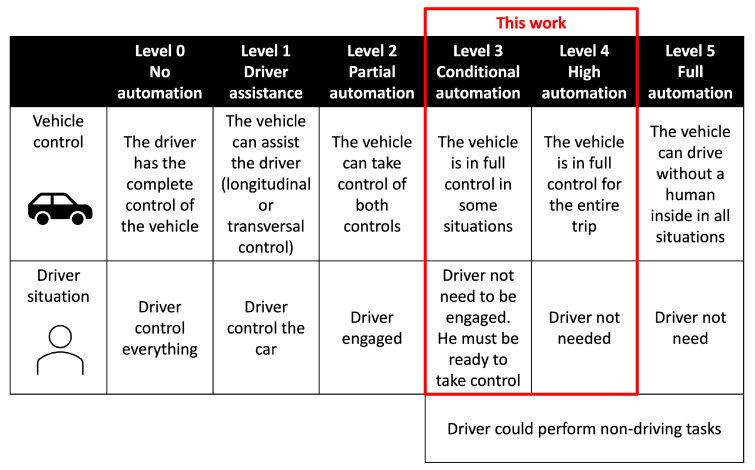
Automation levels, with the level of attention required by the driver.

We situate this work in Levels 3 and 4, where the vehicle is partially autonomous and sometimes requires human interaction at some specific events when the car cannot manage and needs the driver’s intervention. Figure 1 shows the five levels and the different driver engagement requirements to drive safely. In this study, the driver can engage in non-driving-related tasks (NDRTs) and they need a lapse of time to perform the take-over manoeuvre. This lapse begins with the take-over request (TOR) and ends when the driver resumes manual control. The complexity of the driving task and the external conditions (complex scenarios or bad weather conditions) mean that this period has to be long enough to complete the transition safely. To guarantee this requirement, it is mandatory to check whether the driver’s state is suitable for driving. In [2], the authors provide a taxonomy for different forms of autonomous-vehicle handover or take-over. The acknowledgement of the assessment of the driver’s awareness in the driving scenario (e.g., surrounding vehicles or pedestrians) is crucial just before the take-over. This evaluation is usually based on the driver’s visual focalization, predicted by a gaze tracking estimation since the eye gaze is related to human activities [3].

We propose a complete analysis of the driver’s behaviour during the take-over manoeuvre to guarantee a safe transition. This study is complex due to the large amount of data generated during the transition. Drivers perceive the environment by means of their gaze in a short period of time to make the best decision. For this reason, we plan to focus this analysis on four parameters, including the driver’s gaze.

1. Gaze focalization, i.e., the objects at which the driver is focalizing his gaze. We study this behaviour during the transition and the first moments of manual driving after an emergency TOR.
2. Take-over time, the time it takes for the driver to resume manual driving after TOR.
3. Take-over quality, the quality of the driver’s intervention after resuming manual control from the vehicle data (e.g., vehicle velocity, lane position, steering wheel angle, and throttle/brake pedal position).
4. Driver situation awareness (DSA), this novel metric assesses the awareness of the driver during the current traffic situation. DSA appends an additional step concerning the calculation of gaze focalization since it compares the focused objects with those that should have been focused on, according to a supervisor.

We will analyse these parameters in a simulation environment. Several studies have reproduced scenarios using driver simulation environments. They focused their studies on answering how drivers manage take-over manoeuvres by forcing NDRTs just before the transition. Most of these studies comprising take-over time, quality and prediction performance are conducted in experimental scenarios in rural areas with low traffic density. However, driver’s take-over performance still needs to be analysed in stressful traffic use cases in urban scenarios with prolonged NDRT periods before TOR [4].

To meet the above requirements, we will use the open-source CARLA simulator [5] to devise our experiments. It provides urban layouts, multiple vehicle models, pedestrians, traffic signs, etc., which are valuable tools to contrive and validate autonomous driving vehicles, but not for autonomous/manual transitions controlled from a human machine interface (HMI). We took advantage of the facility CARLA provides to build different scenarios. We conducted our experiments in a stressful urban scenario in CARLA, which consisted of an autonomous vehicle approaching a vehicle ahead on a two-lane highway. We provide two use cases: with/without an automobile passing through the left adjacent lane. The TOR is a triggered function of the time to collision (TTC) parameter with the car ahead. Experiments in this scenario aim to study the driver’s ability to resume manual control while he is/is not engaged in NDRTs in the autonomous mode. If the driver does not focalize his gaze on the road in the transition period, the vehicle will perform an emergency break instead of transferring the vehicle control to the user to avoid a collision with the car ahead. This behaviour will be saved for later analysis.

The main contributions to the take-over request study of this paper are: (1) the gaze focalization method based on a camera, which uses OpenFace 2.0 toolkit [6] and our own NARMAX calibration technique [7], and its fusion with a semantic segmentation road image to detect the focalized objects; (2) the development of a challenging take-over urban scenario based on CARLA consisting of transferring the control in an approaching manoeuvre with a vehicle ahead as a function of the TTC; (3) the calculation of a unified framework based on gaze focalization and vehicle data to evaluate driver’s take-over performance in real-time; and (4) the implementation of a novel DSA metric using semantic segmentation and gaze tracking to assess driver’s awareness.

## 2. Related Works

In the literature, there are different studies regarding driver take-over behaviour mainly focused on predicting models using machine-learning approaches and validated using take-over time and take-over quality performance parameters [8,9,10,11,12,13]. Moreover, there are other works focused on the transition study without getting into the modelling of a safety predictor model [14,15,16,17]. All these works show accuracy metrics but do not consider all the variables that influence the driver’s take-over behaviour [18]. Most of these studies are conducted in simulators in rural scenarios with low traffic density [4]. Existing approaches are focused on measuring the reaction time after the TOR signal and they validate their proposals in simulators with less sensory power than the one provided by CARLA or utilise real scenarios with very constrained use cases [19].

To overcome the above limitations, we present a unified framework that analyses the driver’s road-scene understanding through his gaze focalization fused with the powerful sensory system provided by CARLA. Moreover, we propose a novel driver situation-awareness metric next to different take-over times and take-over quality parameters, as performed in other works in the literature. In addition, we developed two stressful urban scenarios based on CARLA which consist of transferring the vehicle control while the ego-vehicle is approaching another vehicle. These scenarios represent dangerous use cases with an imminent collision during a TOR. Performing these kinds of experiments in physical environments would be impossible due to the danger of the situations we want to evaluate. Moreover, the reproducibility of these experiments would be impossible due to the high number of factors that have to be taken into account during the test.

**Figure 2:**
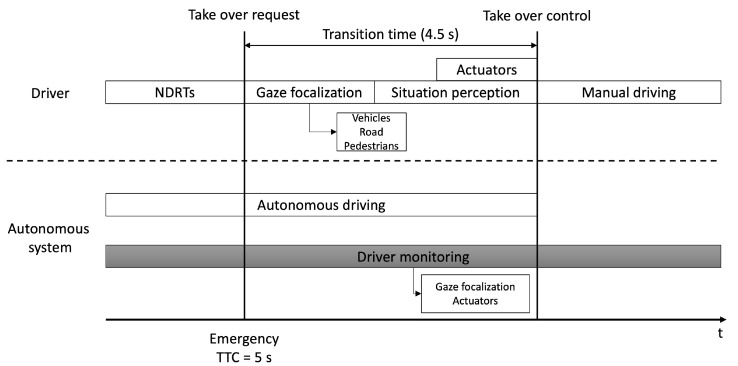
Experiment scenario take-over process timeline.

The designed experiments follow the timeline shown in Figure 2. They begin with the driver engaged or not in a non-driving task during autonomous driving when an emergency occurs (TTC = 5 s). After that, a take-over request is performed, giving the user a transition time (4.5 s). During the transition, the user will have time to gain awareness of the vehicles, pedestrians and other road elements around in order to perceive the situation and get ready to use the actuators to avoid the danger. During the experiment, the module analyses the driver situation in terms of driver perception using gaze focalization and actuators. Finally, at the end of the transition time, it gives the vehicle control to the user. To the best of our knowledge, this is the first time that the hyper-realistic CARLA simulator is used in a take-over study with a transition and fusing the driver’s gaze with the objects of the scene where he is looking in real time. With this fusion, we study the items focalized by the driver during the take-over manoeuvre after a dangerous situation that the autonomous vehicle cannot handle. In addition, this study analyses take-over times and other take-over quality parameters obtained from the vehicle data, such as ego-vehicle velocity, lane position, steering wheel angle, and throttle/brake pedal position.

There are different approaches to performing a TOR. It can be performed via auditory, visual or haptic modalities [20,21,22]. We used a combination of visual and auditory alerts to request the driver reassume the manual control. We implemented both solutions because the driver will perform NDRTs during the experiment and needs both stimuli to get notified. Haptic modalities on the steering wheel can not fit these experiments because the driver is not in contact with the wheel. So he would not sense the warning when he is involved in a NDRT. This last modality is extremely valuable in a TOR that occurs in Level 2, where the driver has to be in contact with the steering wheel.

As mentioned before, this work proposes the analysis of the gaze focalization of the driver during the experiment. For this task, there are different works mainly based on head-mounted eye trackers [23,24,25], active desktop-mounted eye trackers [26] and passive desktop-mounted cameras [3,27,28,29,30]. These approaches captured the human attention but, from our knowledge, the only one that used gaze focalization in a take-over study is [13] using the Tobii Pro-Glasses 2, which is an expensive and intrusive eye tracker. It provides accurate gaze information but uses an intrusive and costly sensor. This work uses the driver’s gaze focalization to feed a deep neural network to predict his take-over behaviour. In our case, we propose the fusion of gaze focalization with the semantic information extracted from a camera placed on the vehicle, which is looking at the road. Merging the gaze vector and the road-scene semantic image, we obtain the object at which the driver is looking. To obtain the gaze, we use our gaze focalization system based on a passive camera, which is a non-intrusive and cheap system. In addition, we use vehicle data synchronized with gaze data.

Using gaze focalization as an awareness metric is valuable because eyes off the road for a time results in a reduced attention level. However, this is just a rough estimation of the driver’s attention and cannot distinguish between the perception of relevant and irrelevant objects inside the driver’s field of view [31].

A new metric is required to measure the driver’s situation awareness to determine his take-over ability to resume manual driving safely. Several studies in the literature assess DSA [32,33], but they do not work in real-time. In [31], authors propose a real-time method inspired by aviation using region-of-interest (ROI) prediction and eye tracking. The comparison of the actual awareness with the target awareness calculates the DSA. Areas on which a driver’s gaze focalizes on objects are the actual awareness and the items where a supervisor thinks the driver should have focalized his gaze are the target awareness. However, this method has only been validated with pure observation, not in challenging take-over scenarios. Our proposal uses semantic segmentation instead of ROIs to determine the target awareness, validated in an urban, stressful transition scenario in real time.

## 3. Experiment Framework

**Figure 3:**
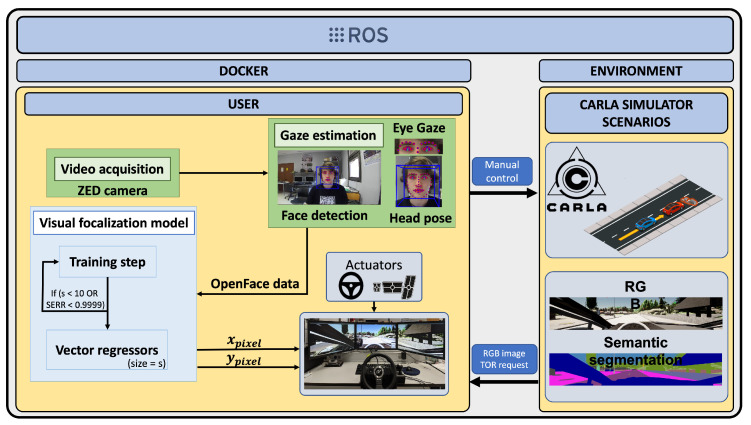
Framework proposed to analyse take-over request (TOR). Green boxes determine the first subsystem/computer and blue boxes the second one.

Our proposal aims to study the driver’s behaviour in take-over manoeuvres using a unified framework based on gaze focalization, scene understanding and vehicle data (velocity, steering wheel, throttle, brake, TTC and lane position). For this task, real-time communication between two data sources (subsystems), the user and the simulator, is needed. Figure 3 shows the framework used in this work. The two subsystems communicate using the robot operating system (ROS) [34] due to the power to distribute processes of ROS. Figure 3 shows the two subsystems in different colours. The first one (user) is in charge of the OpenFace 2.0 toolkit and the user image is monitored using the ZED camera (green boxes). It transmits the information provided by OpenFace 2.0 toolkit using ROS to the second subsystem (simulator) to perform the calibration and run the simulator (blue boxes).

### 3.1. Driver’s Gaze Focalization (Subsystem 1)

Autonomous vehicles (Level 3 onwards) will allow drivers to engage in NDRTs. The assessment of the driver’s visual attention and take-over readiness before the control transfer is crucial. As there is a proven high correlation between the driver’s visual attention and gaze tracking, we will evaluate this parameter during driving. We propose a cheap and non-intrusive gaze focalization method based on a desktop static camera focused on the driver, the OpenFace 2.0 tool [6], and a calibration method using the NARMAX algorithm [35] to evaluate the driver’s gaze. This proposal has been validated in a challenging accidental scenario (DADA2000 [36]) and published by the authors in [7]. The method is satisfactory enough to be an alternative to active desktop-mounted eye trackers or head-mounted eye trackers, which are intrusive, costly and difficult to utilise in physical environments. We obtained on-par results with desktop-mounted eye trackers in similar conditions over the DADA2000 dataset. This method achieves about 1.02% error on the X axis and 1.04% error on the Y axis, which is suitable for the application that we want to test here: the study of the gaze during the transition after a TOR when an emergency occurs.

As stated before, we implemented a distributed system in order to achieve a better performance. The utilisation of two computers is due to the requirements of our simulator (CARLA), which needs most of the computing power to run the world scenario and the driver view on three screens at a reasonable frame rate. We use a simulator to replicate an authentic stressful scenario in a safe environment where accidents can occur with no personal nor material losses. The gaze focalization subsystem, packed in a container using Docker to improve flexibility and compatibility, runs on a computer (subsystem 1) which processes the camera acquisition and the OpenFace 2.0 processing, sending the resulting data as a string by ROS network, which is significantly lighter than an image.

To relate the face-tracking data provided by OpenFace with the areas of the scene where the driver focuses his gaze, each user has to carry out a calibration procedure at the beginning of each experiment. The second subsystem is in charge of this task in addition to running the simulator. The calibration works as follows: firstly, the user has to look at some targets over the simulator screens sequentially for 40 s to obtain the NARMAX training data. This data is unique for each user because the model fits the user under test from scratch. The driver must perform this task as naturally as possible, achieving a model that estimates gaze focalization at 30 fps, the best frame rate according to our ablation study presented on [7]. In total, 1200 samples are obtained per user to train the model. These samples are composed of OpenFace 2.0 data and the corresponding position targets at which the user was looking during the calibration time. Data used from OpenFace 2.0 in this experiment are: Gaze angle X ($\theta_{x}$), Gaze angle Y ($\theta_{y}$), Head position X (*x*), Head position Y (*y*), Head position Z (*z*), Head rotation X ($\Omega_{x}$), Head rotation Y ($\Omega_{y}$), Head rotation Z ($\Omega_{z}$) and Head rotation W ($\Omega_{w}$). All this data references to the camera frame.

The NARMAX model utilises this data for training. It predicts gaze focalization over the simulator screens using the orthogonal least-squares (OLS) algorithm. Once the model is obtained and loaded, the user must perform a quick validation test to verify that the model is working well. The user has to look at the same points as during the training procedure over the three screens. If the gaze projected with the model resembles the test point path, the calibration is over. Otherwise, the user will repeat the calibration process until a correct model is obtained, following the instructions provided by the supervisor.

### 3.2. Simulator (Subsystem 2)

Since the instrumentation of an experimental vehicle has a high cost and conducting experiments on public road networks is difficult because they are highly regulated, a simulation environment is beneficial for developing our take-over experiments before road tests. Moreover, an accident can occur during this kind of experiment, where the driver’s attention is evaluated in a dangerous situation, ensuring that using the simulator is the safest option.

**Figure 4:**
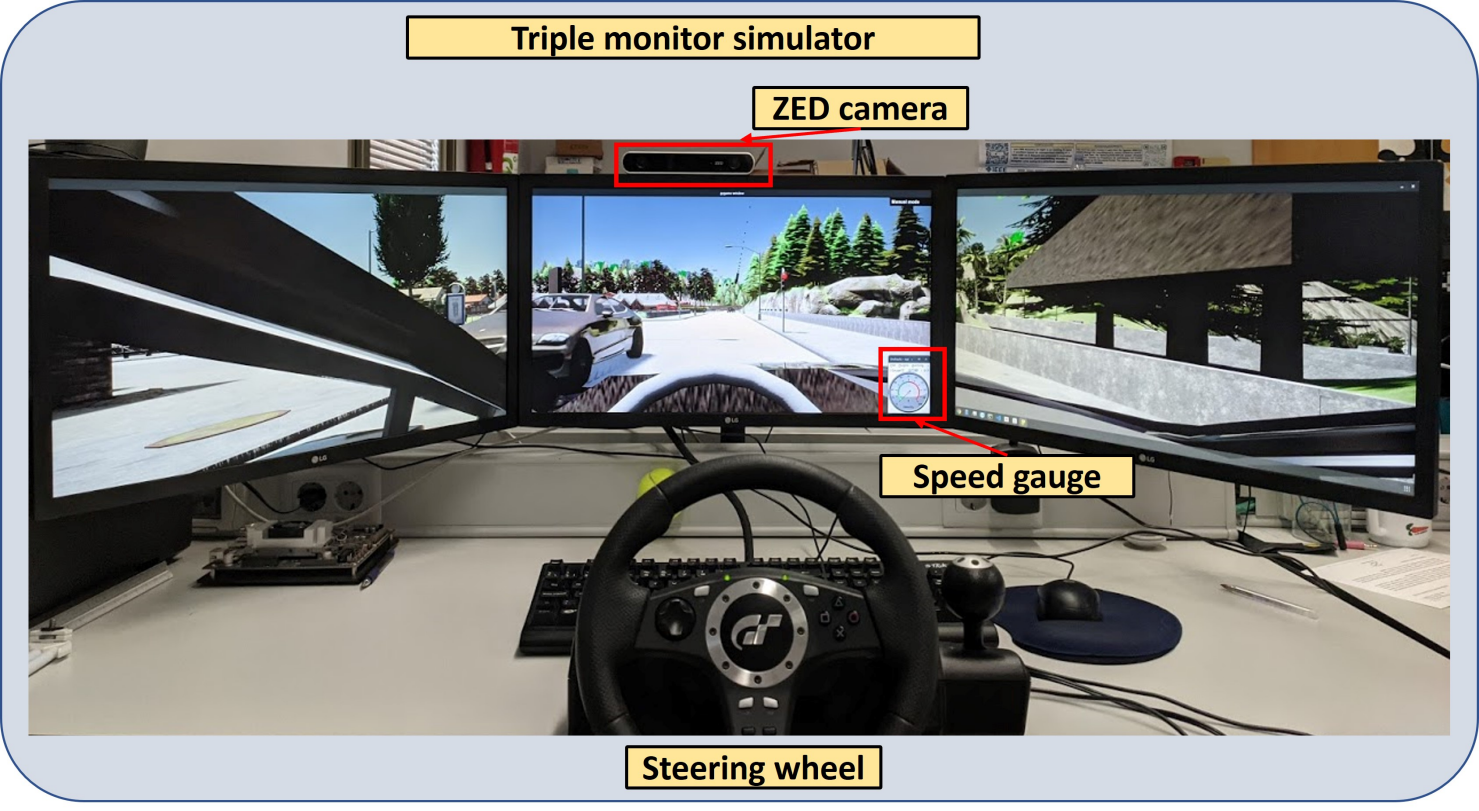
Point of view of the driver during the experiment.

Our simulator comprises three 24-inch screens (full HD resolution 1920 × 1080 p), a steering wheel and pedals (Logitech Driving Force GT) to control the vehicle, and a ZED camera from Stereolabs for gaze tracking. The user performing test is placed in front of the simulator, as shown in Figure 4. This represents the driver’s view during the experiment. The simulator set-up is similar to others used in the literature [37,38], which are composed of three screens and a steering wheel and pedals.

The simulator is based on CARLA [5], which supports the development, training, and validation of autonomous driving systems in a hyper-realistic environment before transferring them to the real world. CARLA is mainly utilised for fully autonomous-driving development. Consequently, we had to adapt the simulator to recreate a real take-over experience, able to shift from autonomous to manual vehicle control. Two challenging scenarios were developed in which users must follow the same route to obtain statistical results. The simulator runs at 13 fps over a computer composed of an Intel i7-9700k (overclocked to 4.9 GHz), NVIDIA 2080 Ti and 32 GB of RAM (3200 MHz). As stated above, this subsystem communication runs simultaneously with the other one using ROS [34]. We published all sensor data provided by CARLA through the ROS bridge developed by CARLA developers. The sensors used in our experiments are: RGB camera and semantic segmentation, as well as some pseudo-sensors such as a Collision sensor, Lane Invasor sensor and vehicle status (velocity, throttle, brake and steering-wheel status). Additionally, custom messages are utilised (e.g., vehicle transition status).

We set both image sensor resolutions (RGB and semantic segmentation) to 5760 × 1080 p. This pixel density is the same as our simulator resolution, which is composed of three screens. These sensors publish images at this resolution to fit the simulator road scene watched from the driver position, allowing to match scene pixels with the gaze vector of the driver. We have set the vehicle camera’s field of view to 100 degrees. It guarantees that the driver has a sight of the road in the simulator similar to the one inside a real vehicle. Figure 4 shows the driver’s view when he is in the simulator. In that figure, we can observe the camera placed on top of the centre screen. This camera is in charge of the gaze focalization. It will have a perfect view of the driver during the experiment.

The objective of this work is to study where drivers focalize their attention during the take-over manoeuvre and their actions during this time. For this reason, we utilised the autopilot provided by CARLA. It offers autonomous driving facilities for the ego-vehicle. It accesses the position of all the objects in the simulated world without compromising the system’s performance to pursue safe driving. By default, the vehicle drives at 70% of the allowed speed in the lane and maintains a safe distance from the vehicle ahead. To perform our stressful take-over scenario, we use the traffic manager, an API that controls the dynamic of all the objects in the CARLA world. We changed it to allow collisions of the ego-vehicle with other agents. In addition, we the speed limit in the lane.

**Figure 5:**
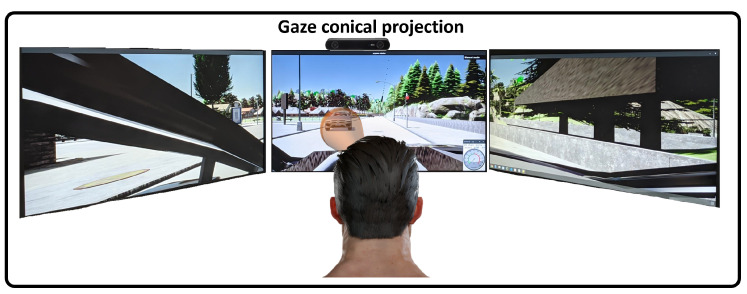
Circular uncertainty gaze area with a radius of 30 pixels on the image following a conical projection represented with an orange cone.

To link gaze focalization to the road scene, we use the semantic-segmentation sensor. It gives a pixel-wise-labelled image with their corresponding classes. This technique has been implemented in the autonomous driving paradigm with different neural networks as ERFNet [39] or DeepLabV3 [40]. Due to the fact that this is not in the scope of this work, we used the one provided by CARLA, which generates the image synthetically from the RGB image. As the sensor has the same resolution as the image displayed on the screens, we project the obtained driver’s gaze vector using the NARMAX calibration model over the semantic-segmentation image, giving us the focalized class. Bearing in mind the error of our focalization system, we project a circular uncertainty gaze area with a radius of 30 pixels on the image following a conical projection (Figure 5). Thus, the segmented object will be the object with most pixels within this area. This approach is similar to the one carried out in [32,41]. They proposed to model the gaze as a cone from the eyes to the windshield.

#### Vehicle Data

Besides the novel study of gaze focalization during the transition presented above, we also measure different vehicle parameters that can be useful for awareness evaluation.

In terms of actuators, we recorded data from the three elements that the user can use during the experiment: the throttle, the brake and the steering wheel. The throttle and the brake are continuous signals which vary from 0 to 1, which means that when the user presses the pedal with all his force, the recorded signal is 1, and if he is not pushing it, it will be 0. Regarding the steering wheel, its signal varies from −1 to 1, meaning that with the steering wheel completely turned to the left, it is −1, and if it is on the opposite side (right), it will be 1.

Another significant parameter to measure is the velocity, as it is responsible for the TOR when an imminent collision with the vehicle ahead is about to happen. We will study the evolution of the above metrics after the TOR to avoid a collision.

Finally, we study the lateral error of the vehicle in the lane. For that, we measure the vehicle’s position inside it and compare the lane with its centre.

## 4. Scenarios

As stated before, evaluation of TOR experiments in challenging situations are scarce in the literature. To overcome this weakness, we propose an experiment in two scenarios on a two-lane highway where the ego-vehicle is driving in autonomous mode. To achieve that, we used the CARLA ScenarioRunner tool [42], which allows the definition of scenarios in CARLA. The module works on Python and uses the OpenSCENARIO standard. This standard aims to describe complex and synchronised manoeuvres. They involve multiple entities, such as vehicles, pedestrians and other traffic participants. The description of a manoeuvre may be based on driver actions (e.g., performing a lane change) or on trajectories (e.g., derived from a recorded driving manoeuvre). In our case, we developed our scenario using the second option, where we defined the agents’ initial and goal positions and velocities. Additionally, we created our test bench for our experiment.

**Figure 6:**
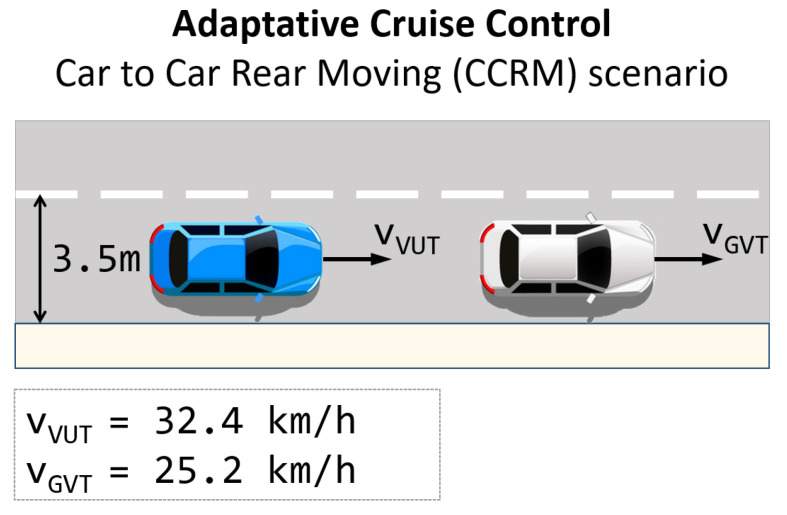
Adaptive cruise control scenario. Ego-vehicle is represented in blue and the other agent in white.

The first scenario is an ACC (adaptive cruise control). In it, the ego-vehicle is following another vehicle. The vehicle ahead is running at 25.2 km/h, and the ego-vehicle, which does not respect the safety distance, is running at 32.4 km/h without decreasing its velocity when it approaches the car ahead. Figure 6 shows the first scenario where the ego-vehicle is in blue, and the adversary is in white. Additionally, other agents in the scenario follow random trajectories, following the traffic rules. The users should perform a breaking manoeuvre.

**Figure 7:**
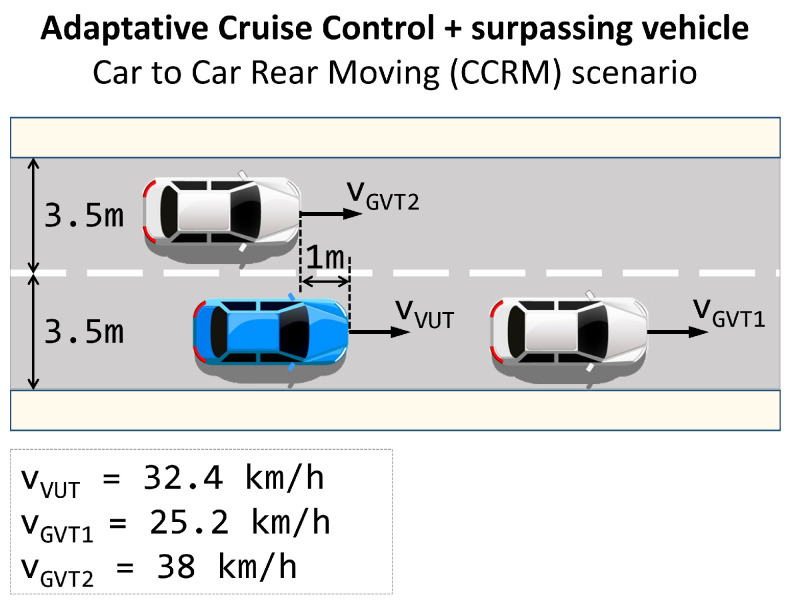
Adaptive cruise control while another vehicle overtakes the ego-vehicle in the side lane scenario. Ego-vehicle is the blue car, and the other agent is the white one.

The second scenario follows Figure 7, where, besides the ACC, another adversary is driving on the left, performing a passing manoeuvre. Vehicle speeds are 25.2 km/h for the one ahead and 38 km/h for the passing vehicle. The ego-vehicle is running at 32.4 km/h. In this scenario, the users cannot perform a lane change because they would collide with the passing vehicle.

The experiment requests the take-over under the same conditions in both scenarios. The trigger is a function of the time to collision (TTC) with the vehicle ahead. Equation (1) calculates it, where *t* is the transition time. We chose this trigger to generate a stressful situation. We set *t* to 4.5 s because [14] claimed that 4.54 s is the mean time a driver needs to take control from autonomous to manual at a simulator.

(1) TTC>t

To obtain the ego-vehicle TTC with each vehicle, we utilise the Constant Turn Rate and Velocity model (CTRV) [43]. It follows the state vector shown in Equation (2), whose next state is calculated according to the non-linear matrix shown on Equation (3). This model predicts the new vehicle position, assuming that the velocity *v* and the yaw rate ω do not correlate.

(2) $$\vec{x}\left(t\right)={\left(\begin{array}{ccccc} x & y & \theta & v & \omega \end{array}\right)}^{T}$$

(3) $$\vec{x}\left(t+T\right)=\left(\begin{array}{c} \frac{v}{\omega }sin\left(\omega T+\theta \right)-\frac{v}{\omega }sin\left(\theta \right)+x\left(t\right)\\ -\frac{v}{\omega }cos\left(\omega T+\theta \right)+\frac{v}{\omega }sin\left(\theta \right)+y\left(t\right)\\ \omega T+\theta \\ v\\ \omega \end{array}\right)$$

We calculate the incoming positions for each vehicle for the next 5 s. Kinematic models without additional rules or semantic information are limited in terms of short-term prediction, commonly in a range of up to 3 s. We do not fulfil this restriction because vehicles do not perform any velocity change during autonomous mode.

**Figure 8:**
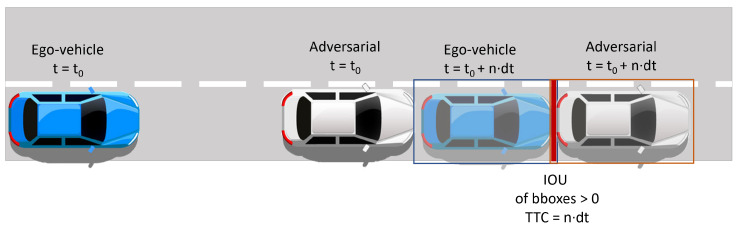
CTRV model prediction and IOU between both vehicles to determine TTC.

With all the vehicle’s positions in the scene image, we obtain a bounding box (bbox) for each vehicle, including the ego-vehicle, and we add a safety distance around the car of 20 cm. We discretized the next 5 s in periods of 0.1 s (dt = 0.1) and predicted the positions of the vehicles using the CTRV model as bbox. With these bounding boxes (bboxes), we calculate the intersection over union (IOU) between the ego-vehicle and the surrounding vehicles for each time step. The first time step when the IOU is more than 0 corresponds with the TTC of that vehicle. Figure 8 shows this procedure.

## 5. Experiment Setup

For this experiment, we requested 20 drivers (15 men and 5 women) to perform the test. They were required to have their driving licence and not have ever seen the scenario before. Before the experiment, we informed the users of the objective of the study. Their mean age was 33.5, with a standard deviation of 11.9 years. They had a mean possession of a driving licence of 15.3 years with a standard deviation of 12.1 years. We conducted this experiment in Spain, where the minimum age to obtain a driving licence is 18 years old.

When a dangerous situation (TTC = 5 s) takes place, the vehicle will warn the user and give him a transition time of 4.5 s to prepare to take the car’s controls. A driver’s behaviour study in this interval is crucial to ensure a safe control transition. This warning (TOR) is received by the user in two different ways, as a sound and warning message on the screen, which indicate that in the next 4.5 s the car will change its navigation mode.

During the transition time, if the user does not focalize his gaze on the scene because he is engaged in an NDRT, the car will make an emergency break and will give him control when the user’s gaze is correct. If this happens, we record the sequence for posterior analysis. We recorded every user and vehicle data in a text file to analyse each user and obtain statistical results.

Before the experiment begins, the user has to perform some steps: firstly, the user has to calibrate the gaze focalization system. With the calibrated system, the user will perform some routes using the simulator to get used to it. As it is the first time they are in the simulator, they need to feel confident about using it. Finally, with the tool calibrated and the user confident with in the simulator, the experiment can begin with the two scenarios.

## 6. Results

The presented results correspond to a study with 20 users in a TOR experiment conducted in our simulator based in CARLA. We studied the fusion between gaze focalization and the semantic segmentation provided by the CARLA simulator after a TOR when an emergency happens during an autonomous driving simulation. Moreover, we studied the driver’s reaction times in terms of the actuators used, as other works have done in the literature. Finally, to understand the situation of the driver and their ability to resume driving, we introduce driver situation awareness (DSA), a novel metric presented in [31]. We use it to measure driver awareness after the TOR. The aviation field uses situation awareness (SA) [44] to measure the pilots’ attention level. Table 1 summarises the Results chapter.

### 6.1. Gaze Focalization Analysis

The principal analysis of this work is the study of gaze focalization during the transition in some users. For this purpose, we present two different results for our TOR study: the fusion between the gaze and the semantic segmentation and the use by the user of the different screens of our simulator, which is composed of three screens.

#### 6.1.1. Fusion of the Gaze Focalization and the Semantic Segmentation

The fusion between the driver’s gaze focalization and the semantic segmentation during the transition gives information about his behaviour. To summarise the results, we split the experiment into three sections (manual, autonomous and transition), which are the different driving modes the vehicle will find during the route. Matching the gaze and the semantic information will help to understand the driver’s awareness.

**Figure 9:**
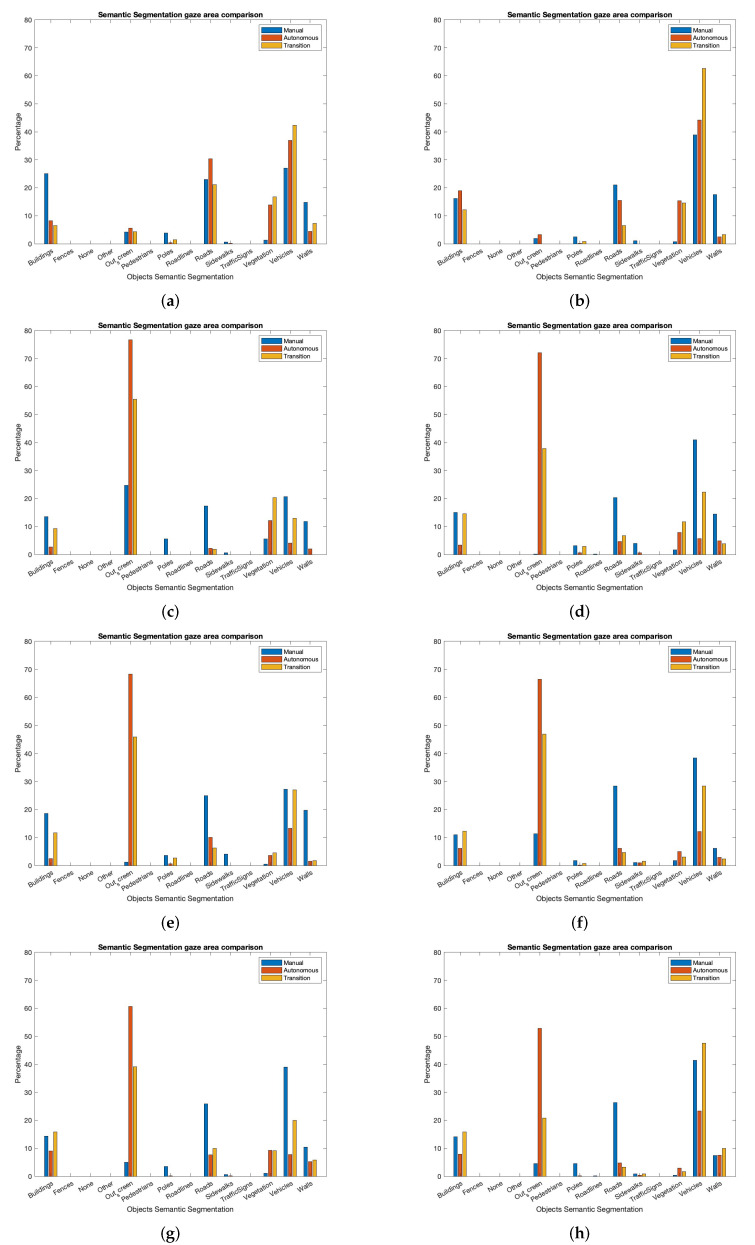
Gaze focalization analysis with semantic segmentation. (**a**) Experiment: ACC. Driver attention: focus. (**b**) Experiment: ACC + passing. Driver attention: focus. (**c**) Experiment: ACC. Driver attention: using the mobile phone. (**d**) Experiment: ACC + passing. Driver attention: using the mobile phone. (**e**) Experiment: ACC. Driver attention: reading. (**f**) Experiment: ACC + passing. Driver attention: reading. (**g**) Experiment: ACC. Driver attention: talking to passenger. (**h**) Experiment: ACC + passing. Driver attention: talking to passenger.

The driver focuses his gaze on different items according to the task he is involved in before the TOR. This analysis can be seen in Figure 9. It shows the percentage of focalized objects for the three different driving modes. We split the figure into two columns, one for each scenario, and four rows, one for each driving behaviour before the TOR. The left column shows the ACC use case and the right column the ACC with a passing vehicle use case. The first row (a) shows the case in which the user is focused on the autonomous driving. The second row (b) shows the experiment in which the user uses his mobile phone during autonomous driving. The third row (c) shows the experiment with the user reading before the TOR. Finally, the fourth row (d) shows the experiment with the user conversing with the co-pilot.

In these figures, we can appreciate the object where the driver is focusing his gaze thanks to the fusion with semantic segmentation. According to Figure 9, with the vehicle in autonomous mode and the user involved in monitoring the driving, they focalize their gaze on cars, roads and buildings. It means that they are aware of the driving situation. However, when they are involved in a non-driving-related task, their gaze is not focused on any object because they are not monitoring the driving.

After the autonomous driving mode, we trigger the transition mode. According to the obtained result, when the user monitors the driving, he keeps focalizing on the same objects as in the previous section (autonomous driving mode). That means that the driver is involved in the driving task even if he is not driving, and he will be able to resume control by being aware of the situation.

If the driver is involved in non-driving related tasks, he needs more time to become aware of the situation. For this reason, the most watched category during the transition is “out of the screen”. It means that he does not focalize his gaze on the simulator and, therefore, he is out of the loop. The reason for this result is that the driver is not aware of the driving and needs time to place their gaze on the simulation scene after the request to resume control. After focalizing the gaze on the simulator, the most watched category is “vehicles”. The driver feels that the cars are the most relevant objects to be aware of ahead of others on the scene to avoid the collision.

Finally, after the transition, the vehicle will change to manual mode if it detects that the driver has focalized his gaze on the road. Therefore, the ego-vehicle will be in manual mode, and the driver will have to drive. During these experiments, if the vehicle is in manual mode, the most watched category is “vehicles”. Most of the users under the test focalized their gaze on the vehicle ahead to keep performing an ACC.

Besides Figure 9, in which the different gaze focalizations during all the modes are shown graphically, Table 2 shows the same results but expressed quantitatively. This table shows the result for the three split modes.

During manual mode, the driver focuses his gaze on the scene. As he is driving, the most watched classes are “vehicles”, “roads”, and “buildings”. These categories represent the most relevant classes in the designed scenarios. During the autonomous mode, the user carries out non-driving tasks for 75 per cent of the experiments (3 of 4). In the remaining 25 per cent (1 of 4), the user focuses his gaze on the scene when the vehicle is in autonomous mode to show a comparison between monitoring or not driving. Nevertheless, some users looked at the scene while they talked to another user, when performing the conversation with the co-pilot experiment. Due to that, the most watched category is “out of the screen”, followed by “vehicles” and “walls”.

Finally, during the transition, the most watched category is “vehicles”, followed by “out of screen” because the user is not involved in the driving, but their reaction time is good enough to focus their gaze on the scene, as will be shown later.

These results show the same classes for the different modes with slight differences depending on the driving mode. During manual driving, the most focused class are “vehicles” and “roads”. The driver is aware of the scene and does not to collide with the vehicles ahead. However, he also has time to look at the environment (“buildings”). In autonomous mode, the driver does not need to be aware of the scene because he is involved in other tasks. In the transition, the driver focalizes on the classes “vehicles” and “out of the screen”. He needs time due to the gaze reaction time.

#### 6.1.2. Analysis of the Gaze Focalization on the Different Screens

Gaze semantic information about the object watched by the users during each section of the experiment is our principal analysis for the TOR study regarding the gaze. Nevertheless, we want to present the results of the use of the simulator in terms of gaze focalized over the different screens for the transition.

Table 3 shows the screen percentage focalized on during the transition after the TOR. Results show that if the driver is not performing any non-driving task, he will focalize on the centre screen because it displays the principal object that should be focused on (the vehicle ahead).

### 6.2. Vehicle Status Analysis

After the presentation of the novel results exposed in this work focused on driver’s gaze focalization fused with the semantic segmentation, we will highlight some variables regarding the vehicle and actuators that influence this study.

#### 6.2.1. Vehicle Velocity Analysis during the Transition

**Figure 10:**
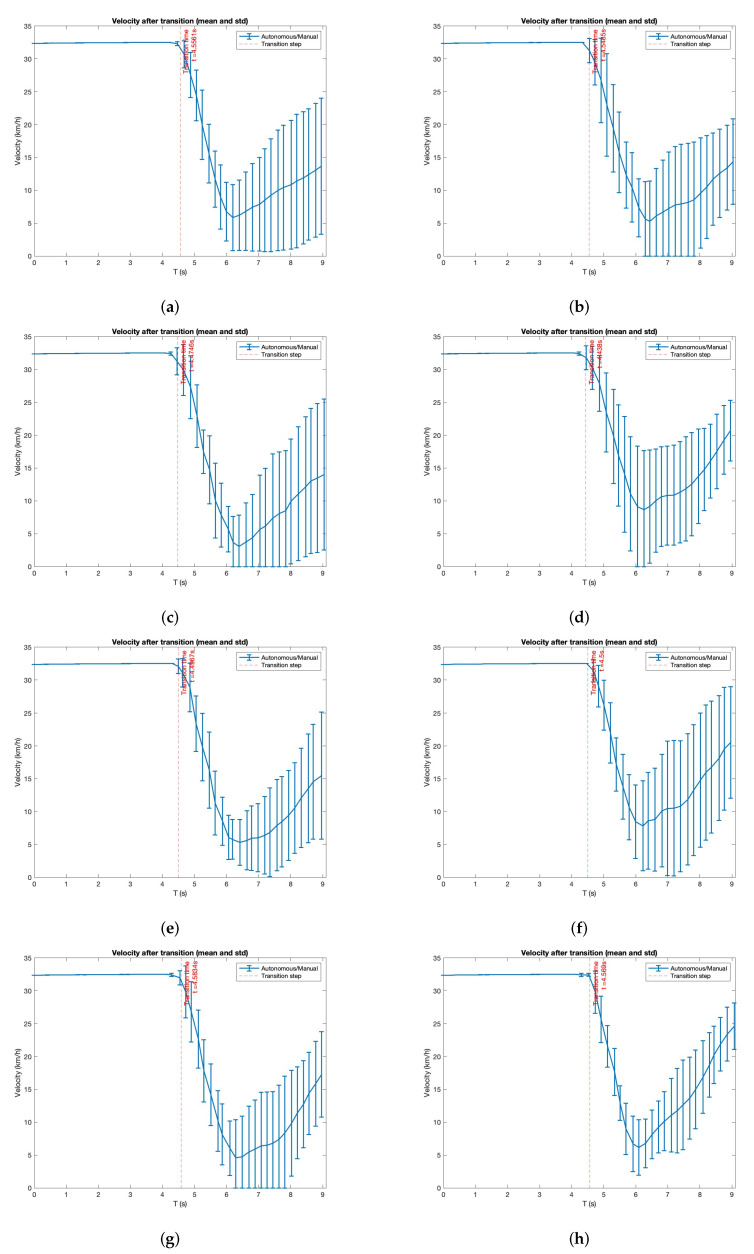
Velocity analysis after transition (mean and standard deviation). Transitions are split in autonomous to manual. (**a**) Experiment: ACC. Driver attention: focus. (**b**) Experiment: ACC + passing. Driver attention: focus. (**c**) Experiment: ACC. Driver attention: using the mobile phone. (**d**) Experiment: ACC + passing. Driver attention: using the mobile phone. (**e**) Experiment: ACC. Driver attention: reading. (**f**) Experiment: ACC + passing. Driver attention: reading. (**g**) Experiment: ACC. Driver attention: talking to passenger. (**h**) Experiment: ACC + passing. Driver attention: talking to passenger.

The first variable is the velocity of the vehicle (Figure 10). The schema of the figure is the same as the previous analysis, where columns separate both scenarios and rows split the different driver behaviours. In this figure, we can see the velocity after the TOR. The graphics show 4.5 s of transition mode followed by 4.5 s of manual mode. We do not display autonomous mode because it is not representative. It shows the mean and standard deviation for all the users. The vertical red line represents the transition moment where the user resumes vehicle control.

As expected, the velocity decreases just after the transition to avoid a collision with the vehicle ahead. As we trigger the TOR when the TTC with that vehicle is lower than a threshold, users decrease the velocity after the transition when they have vehicle control. We will analyse this behaviour during the actuators’ study. There are only slight differences between the experiments in this study. The most evident one (see Figure 10) is that performing the same non-driving task is faster in the first experiment (ACC with the vehicle passing) than in the ACC one. The reason for this is that users conduct these experiments after the ACC, and they feel more confident with the simulator.

#### 6.2.2. Vehicle Actuators Analysis during the Transition

**Figure 11:**
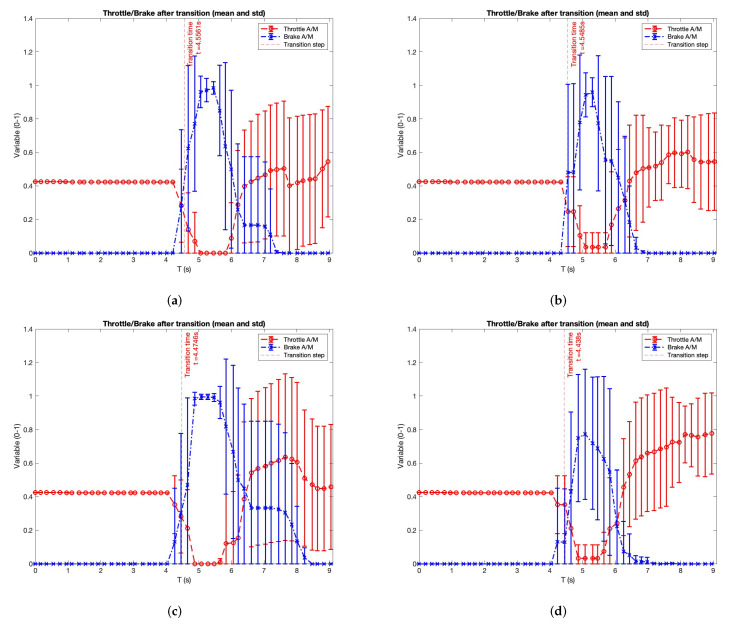

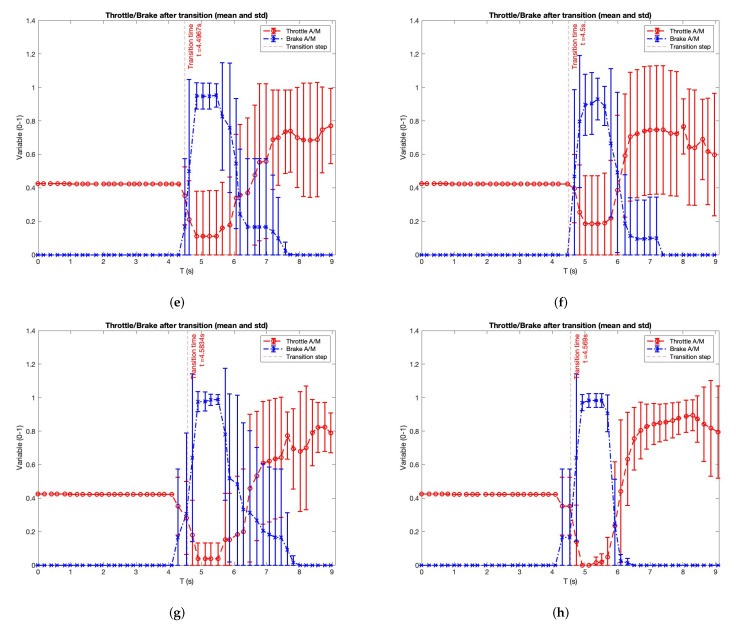
Throttle and brake analysis after transition (mean and standard deviation). Transitions are split into autonomous to manual (A/M). (**a**) Experiment: ACC. Driver attention: focus. (**b**) Experiment: ACC + passing. Driver attention: focus. (**c**) Experiment: ACC. Driver attention: using the mobile phone. (**d**) Experiment: ACC + passing. Driver attention: using the mobile phone. (**e**) Experiment: ACC. Driver attention: reading. (**f**) Experiment: ACC + passing. Driver attention: reading. (**g**) Experiment: ACC. Driver attention: talking to passenger. (**h**) Experiment: ACC + passing. Driver attention: talking to passenger.

**Figure 12:**
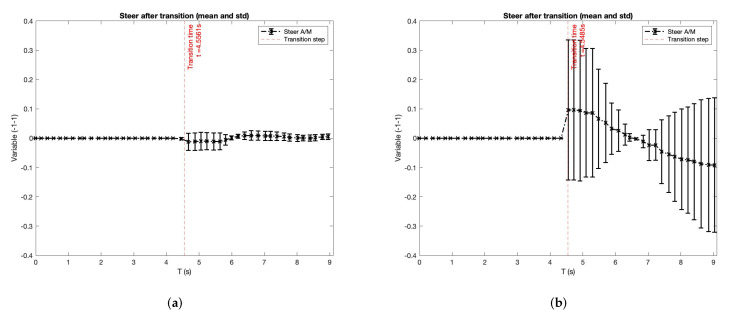

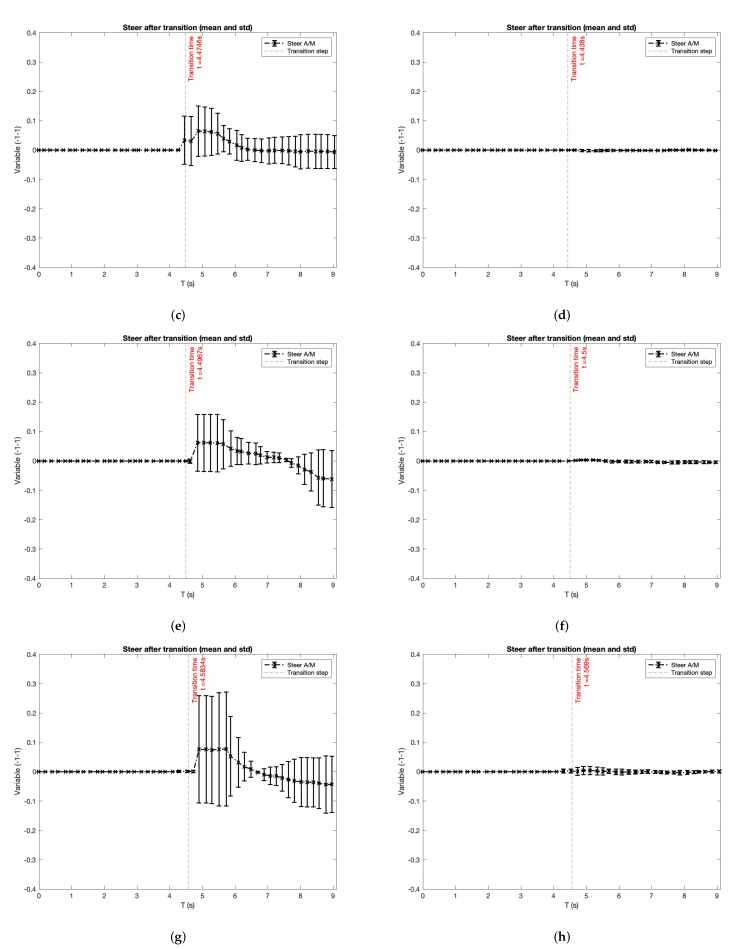
Steer analysis after transition (mean and standard deviation). Transitions are splitted in Autonomous to Manual (A/M). (**a**) Experiment: ACC. Driver attention: focus. (**b**) Experiment: ACC + passing. Driver attention: focus. (**c**) Experiment: ACC. Driver attention: using the mobile phone. (**d**) Experiment: ACC + passing. Driver attention: using the mobile phone. (**e**) Experiment: ACC. Driver attention: reading. (**f**) Experiment: ACC + passing. Driver attention: reading. (**g**) Experiment: ACC. Driver attention: talking to passenger. (**h**) Experiment: ACC + passing. Driver attention: talking to passenger.

Velocity is a metric that gives a good understanding of the use of the actuators, but an analysis of them is necessary to complement this study. Figure 11 shows the analysis of the throttle and the brake in the same way as performed with the velocity. Moreover, Figure 12 shows the analysis of the steering wheel during the experiment with the same layout.

These experiments show interesting conclusions about users’ behaviour in the actuators. In the ACC-mobile, ACC-reading, ACC-talking and ACC + passing-talking cases, users utilised the brake with slight differences between them just after the transition, showing a standard deviation of less than 10 per cent. The remaining cases (ACC-focus, ACC + passing-mobile, ACC + passing-reading, ACC + passing-talking) show more differences in the breaking use. The reason for this behaviour is that the mean value is near 1. It means that the user has pressed the brake with all his strength.

Throttle analysis shows a standard behaviour in all the experiments: users did not use the throttle after the transition’s end because of the imminent collision situation. After that event, the users utilise the throttle to continue the route.

The steering-wheel analysis presented in Figure 12 does not give many conclusions because users did not use it in a rough way to avoid a collision. They felt more self-confident with the brake and thought they have enough distance to perform an emergency brake instead of a manoeuvre to dodge the other car. In addition, this manoeuvre will be impossible to execute in the second scenario because there is an adversarial car passing.

#### 6.2.3. Vehicle Lane-Error Analysis during the Transition

**Figure 13:**
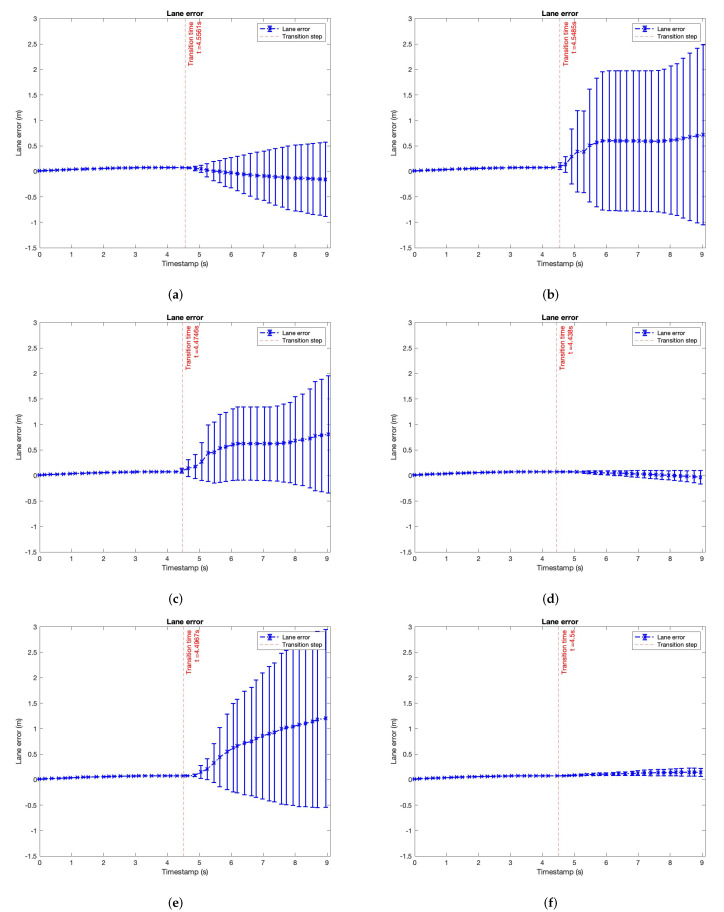

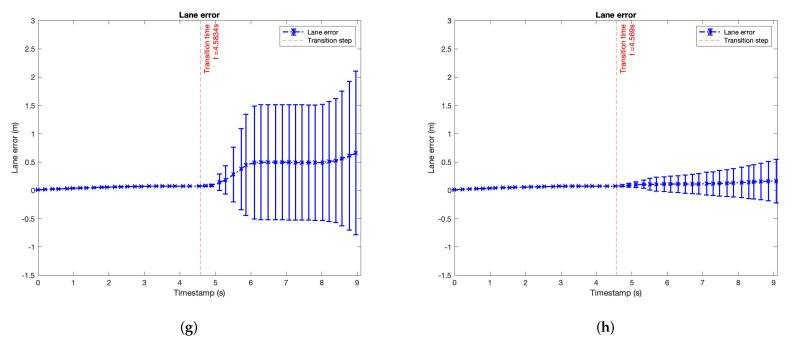
Lane error analysis after transition (mean and standard deviation). Transitions are splitted in Autonomous to Manual (A/M). (**a**) Experiment: ACC. Driver attention: focus. (**b**) Experiment: ACC + passing. Driver attention: focus. (**c**) Experiment: ACC. Driver attention: using the mobile phone. (**d**) Experiment: ACC + passing. Driver attention: using the mobile phone. (**e**) Experiment: ACC. Driver attention: reading. (**f**) Experiment: ACC + passing. Driver attention: reading. (**g**) Experiment: ACC. Driver attention: talking to passenger. (**h**) Experiment: ACC + passing. Driver attention: talking to passenger.

To end with the analysis focused on the vehicle status, we explore the lane error, measured as the displacement of the ego-vehicle position to the centerline of the lane in meters. Figure 13 represents it. This analysis is related to the use of the steering wheel to keep the vehicle centred in the lane.

A constant increase in the lane error means that the trajectory is wrong because the user has not corrected this with the steering wheel. It occurs in the first experiment when the driver monitors the driving. The error increases constantly, correlating to Figure 12, where the users slightly used the steering wheel during the recorded time.

### 6.3. Reaction-Time Analysis

This section shows the relationship between the driver and time. We will use the literature metrics about reaction times in the actuators, contributing to the reaction times of gaze focalization.

Table 4 presents the mean for all the users. The first three columns are the reaction times in terms of gaze focalization. It is the average time the users need to focalize the three most representative objects. These object classes are “roads”, “vehicles”, and “screen”. The three last columns show the actuators’ reaction times (throttle, brake and steering wheel).

Gaze-focalization reaction times show that the users firstly pay attention to the screen because they need to be aware of the scene after performing a non-driving task, with a mean value of 0.86 s for all the experiments. After that, they focalized on the vehicle ahead, with a mean value of 1.96 s, and continued with the brake use, with a mean value of 1.97 s. This sequence is logical for this kind of experiment, with only the absolute values changing depending on the distraction modes.

In the ACC experiment, the variations are due to doubts about breaking or performing an overtaking manoeuvre to avoid the collision. The more distracted the driver is, the longer it takes to conclude that he must execute a braking manoeuvre. It causes an initial steering wheel turn manifested as a lateral lane displacement. Neverthless, the passing use case is more stressful. When the driver focuses his attention on the driving, he doubts whether to overtake. However, when he sees a passing car, he becomes scared and swerves in the opposite direction, executing a braking manoeuvre. When he is distracted, he does not have time to think about the best manoeuvre and simply brakes to avoid crashing.

### 6.4. Situation Awareness

Finally, we add a metric for measuring the driver’s situation awareness (SA) to determine his take-over ability, as claimed in [31]. Most driver’s take-over behaviour metrics in the state of the art do not consider the environment and what the driver is aware of from the surrounding elements. However, a better understanding of the driver behaviour and context is fundamental for these systems to verify if the driver is aware of relevant objects before passing him the control. Methods used in aviation to test pilots’ awareness inspired this use. The authors of [44] defined the SA score as the ratio of the actual SA and the optimal SA. Equation (4) shows that score, where $SA_{actual}$ is the actual situation awareness and $SA_{optimal}$ is the expected situation awareness.

(4) $$SA_{ratio}=\frac{SA_{actual}}{SA_{optimal}}$$

The optimal SA defines awareness as the situation where the driver perceives and comprehends all situation elements (SEs) as the weighted sum of required ($SE_{opt,r}$) elements and desired ($SE_{opt,d}$) elements. In our case, the SEs are obtained automatically and online through semantic segmentation, considering the relevant elements for driving (vehicles, pedestrians, cyclists, traffic lights, etc.) in the scene.

(5) $$SA_{optimal}=2\sum_{r=1}^{m}\;\cdot \;SE_{opt,r}+\sum_{d=1}^{n}\;\cdot \;SE_{opt,d}$$

Equation (5) shows the optimal SA where the difference between required and desired elements is calculated as a function of their Euclidean distance to the ego-vehicle.

We calculate the actual SA using Equation (6), where $p_{drt}$ and $p_{irt}$ are 0 for an undetected SE, 0.5 for a detected SE, and 1 for a comprehended SE, to differentiate between these detection levels.We implemented a time window to measure the gaze over the different objects. Nevertheless, in contrast to the original work, we used semantic segmentation instead of ROIs over the detections because it gives more accuracy than an ROI due to the former technique using pixel-wise labelling.

(6) $$SA_{actual}=2\sum_{r=1}^{m}\;\cdot \;SE_{act,r}+\sum_{d=1}^{n}\;\cdot \;SE_{act,d}=2\sum_{r=1}^{m}\;\cdot \;p_{irt}+\sum_{d=1}^{n}\;\cdot \;p_{drt}$$

Table 5 shows the situation awareness ratio results. There is a clear difference between the experiments with the user focused on driving and the experiments where the user performs a non-driving task for the two scenarios. The values are higher in the most stressful scenario and, therefore, require a higher level of awareness. However, the most distracting task is the mobile in both cases, with reading and talking at an intermediate level. These conclusions are in-line with what was expected and, therefore, validate the use of this metric in the context of the driver take-over behaviour.

## 7. Conclusions

We presented a take-over request study with novel contributions to the literature. These contributions follow the use of our gaze focalization system to evaluate the driver’s behaviour. We analysed the transition between manual and autonomous modes using state-of-the-art metrics and a novel DSA metric for 20 users evaluated in two scenarios where they were performing non-driving tasks.

Experiments concluded that after transition, users focalize their gazes on vehicles and perform a sequence according to the situation proposed in the experiments, aiming to avoid a collision with the car ahead. Slight differences depending on the NDRTs are shown in gaze focalization, showing that during the transition, the driver was more focused on the vehicles and the road than the environment, which is understandable for safe driving.

Vehicle status analysis shows that users decreased the ego-vehicle velocity just after take-over to avoid a collision with the vehicle ahead. The reaction times analysis shows that users were ready to drive after a 4.5 s transition because they comprehended the scene and used the brake even before the transition has finished. Moreover, the situation-awareness metric for these scenarios (DSA) shows that users were aware of the situation most of the time during the transition. The DSA analysis shows that when the drivers were using their phones, they achieved the lowest DSA, inducing that this experiment option is the most distracting of the ones proposed here. Our study shows that DSA correlates with user actions.

The authors want to underline that the sample used in this work is limited, and conclusions obtained may not be generalizable. This study is a proof of concept of an intensive study that should follow the metrics and evaluations presented here. However, results show similar trends to other state-of-the-art studies, which validate them.

In future works, we plan to widen the number of drivers in different age groups and the number of scenarios on CARLA. We want to perform this study in the real world using an autonomous vehicle. Additionally, we plan to develop a predicting model to know if the driver will be aware of the situation to resume control after a TOR to carry it out safely.

## Figures and Tables

**Table 1:** Results summary agenda.

Behaviour Studied	Section	Figure/Table
Gaze focalization object analysis	Section 6.1.1	Figure 9 and Table 2
Gaze-focalization screen analysis	Section 6.1.2	Table 3
Velocity behaviour analysis	Section 6.2.1	Figure 10
Throttle and brake behaviour analysis	Section 6.2.2	Figure 11
Steer behaviour analysis	Section 6.2.2	Figure 12
Lane-error behaviour analysis	Section 6.2.3	Figure 13
Reaction-time behaviour analysis	Section 6.3	Table 4
Driver situation awareness (DSA) analysis	Section 6.4	Table 5

**Table 2:** Gaze focalization analysis with semantic segmentation.

Gaze Median Area			
	**First Class**	**Second Class**	**Third Class**
Manual	Vehicles (36.17%)	Roads (23.75%)	Building (13.07%)
Autonomous	Out of screen (52.68%)	Vehicles (23.37%)	Walls (8.12%)
Transition	Vehicles (47.5%)	Out of screen (20%)	Walls (12.5%)

**Table 3:** Gaze focalization percentage over the three screens during the transition after the take-over request.

Use Case	Distraction	Screen Analysis			
		**Left**	**Center**	**Right**	**Out of Screen**
ACC	Focus	0.00%	100.00%	0.00%	0.00%
ACC	Mobile	3.59%	72.66%	5.75%	17.98%
ACC	Reading	1.43%	90.64%	3.59%	4.31%
ACC	Talking	6.47%	78.41%	10.07%	5.03%
ACC + passing vehicle	Focus	0.00%	100.00%	0.00%	0.00%
ACC + passing vehicle	Mobile	1.44%	74.63%	4.34%	19.56%
ACC + passing vehicle	Reading	11.51%	76.25%	7.19%	5.03%
ACC + passing vehicle	Talking	0.00%	84.89%	7.91%	7.19%

**Table 4:** Reaction time for the different experiments performed.

		Reaction Time (s)					
**Use Case**	**Distraction**	**Roads**	**Vehicle**	**Screen**	**Throttle**	**Brake**	**Steer**
ACC	Focus	3.08	0.13	0.00	3.22	2.01	3.21
ACC	Mobile	7.07	2.93	1.34	1.88	1.69	2.29
ACC	Reading	2.11	3.56	1.23	1.87	1.87	2.95
ACC	Talking	3.13	2.73	1.13	4.44	2.33	4.37
ACC + passing vehicle	Focus	4.69	0.86	0.00	5.41	1.66	2.66
ACC + passing vehicle	Mobile	4.36	1.97	1.08	4.70	2.08	2.25
ACC + passing vehicle	Reading	3.78	2.22	1.47	5.24	2.24	2.24
ACC + passing vehicle	Talking	3.64	1.31	0.65	4.29	1.85	2.31

**Table 5:** Situation-awareness ratio for the different experiments performed.

Use Case	Distraction	Awareness Ratio Transition
ACC	Focus	0.6131
ACC	Mobile	0.287
ACC	Reading	0.4144
ACC	Talking	0.3583
ACC + Passing vehicle	Focus	0.7602
ACC + Passing vehicle	Mobile	0.4272
ACC + Passing vehicle	Reading	0.4308
ACC + Passing vehicle	Talking	0.6958

## Data Availability

Not applicable.

## References

[1] Jimenez F. (2017). Intelligent Vehicles: Enabling Technologies and Future Developments.

[2] McCall R., McGee F., Mirnig A., Meschtscherjakov A., Louveton N., Engel T., Tscheligi M. (2019). A taxonomy of autonomous vehicle handover situations. Transp. Res. Part A Policy Pract..

[3] Yang L., Dong K., Dmitruk A.J., Brighton J., Zhao Y. (2019). A Dual-Cameras-Based Driver Gaze Mapping System with an Application on Non-Driving Activities Monitoring. IEEE Trans. Intell. Transp. Syst..

[4] Santos G.H.G., Larocca A.P.C. Drivers Take-Over Performance from Partial Automation to Manual Driving. Proceedings of the 2019 33th Congresso de Pesquisa e Ensino em Transporte da ANPET.

[5] Dosovitskiy A., Ros G., Codevilla F., Lopez A., Koltun V. (2017). CARLA: An open urban driving simulator. arXiv.

[6] Baltrusaitis T., Zadeh A., Lim Y.C., Morency L.P. Openface 2.0: Facial behavior analysis toolkit. Proceedings of the 2018 13th IEEE International Conference on Automatic Face & Gesture Recognition (FG 2018).

[7] Araluce J., Bergasa L.M., Ocaña M., López-Guillén E., Revenga P.A., Arango J.F., Pérez O. (2021). Gaze Focalization System for Driving Applications Using OpenFace 2.0 Toolkit with NARMAX Algorithm in Accidental Scenarios. Sensors.

[8] Berghöfer F.L., Purucker C., Naujoks F., Wiedemann K., Marberger C. Prediction of take-over time demand in conditionally automated driving-results of a real world driving study. Proceedings of the Human Factors and Ergonomics Society Europe.

[9] Lotz A., Weissenberger S. (2018). Predicting take-over times of truck drivers in conditional autonomous driving. International Conference on Applied Human Factors and Ergonomics.

[10] Deo N., Trivedi M.M. (2019). Looking at the driver/rider in autonomous vehicles to predict take-over readiness. IEEE Trans. Intell. Veh..

[11] Du N., Zhou F., Pulver E., Tilbury D., Robert L.P., Pradhan A.K., Yang X.J. Predicting Takeover Performance in Conditionally Automated Driving. Proceedings of the 2020 CHI Conference on Human Factors in Computing Systems.

[12] Du N., Zhou F., Pulver E.M., Tilbury D.M., Robert L.P., Pradhan A.K., Yang X.J. (2020). Predicting driver takeover performance in conditionally automated driving. Accid. Anal. Prev..

[13] Pakdamanian E., Sheng S., Baee S., Heo S., Kraus S., Feng L. Deeptake: Prediction of driver takeover behavior using multimodal data. Proceedings of the 2021 CHI Conference on Human Factors in Computing Systems.

[14] Eriksson A., Banks V., Stanton N. (2017). Transition to manual: Comparing simulator with on-road control transitions. Accid. Anal. Prev..

[15] Merat N., Jamson A.H., Lai F.C., Daly M., Carsten O.M. (2014). Transition to manual: Driver behaviour when resuming control from a highly automated vehicle. Transp. Res. Part F Traffic Psychol. Behav..

[16] Du N., Zhou F., Pulver E.M., Tilbury D.M., Robert L.P., Pradhan A.K., Yang X.J. (2020). Examining the effects of emotional valence and arousal on takeover performance in conditionally automated driving. Transp. Res. Part C Emerg. Technol..

[17] Ebnali M., Hulme K., Ebnali-Heidari A., Mazloumi A. (2019). How does training effect users’ attitudes and skills needed for highly automated driving?. Transp. Res. Part F Traffic Psychol. Behav..

[18] Zeeb K., Buchner A., Schrauf M. (2015). What determines the take-over time? An integrated model approach of driver take-over after automated driving. Accid. Anal. Prev..

[19] Rangesh A., Deo N., Greer R., Gunaratne P., Trivedi M.M. (2021). Autonomous Vehicles that Alert Humans to Take-Over Controls: Modeling with Real-World Data. arXiv.

[20] Naujoks F., Mai C., Neukum A. (2014). The effect of urgency of take-over requests during highly automated driving under distraction conditions. Adv. Hum. Asp. Transp..

[21] Pakdamanian E., Feng L., Kim I. (2018). The effect of whole-body haptic feedback on driver’s perception in negotiating a curve. Proceedings of the Human Factors and Ergonomics Society Annual Meeting.

[22] Wan J., Wu C. (2018). The effects of vibration patterns of take-over request and non-driving tasks on taking-over control of automated vehicles. Int. J. Hum. Comput. Interact..

[23] Dalmaijer E., Mathôt S., Stigchel S. (2013). PyGaze: An open-source, cross-platform toolbox for minimal-effort programming of eyetracking experiments. Behav. Res. Methods.

[24] Cognolato M., Atzori M., Müller H. (2018). Head-mounted eye gaze tracking devices: An overview of modern devices and recent advances. J. Rehabil. Assist. Technol. Eng..

[25] Shen J., Zafeiriou S., Chrysos G.G., Kossaifi J., Tzimiropoulos G., Pantic M. The First Facial Landmark Tracking in-the-Wild Challenge: Benchmark and Results. Proceedings of the 2015 IEEE International Conference on Computer Vision Workshop (ICCVW).

[26] Xia Y., Zhang D., Kim J., Nakayama K., Zipser K., Whitney D. Predicting driver attention in critical situations. Proceedings of the Asian Conference on Computer Vision.

[27] Mizuno N., Yoshizawa A., Hayashi A., Ishikawa T. Detecting driver’s visual attention area by using vehicle-mounted device. Proceedings of the 2017 IEEE 16th International Conference on Cognitive Informatics & Cognitive Computing (ICCI* CC).

[28] Vicente F., Huang Z., Xiong X., De la Torre F., Zhang W., Levi D. (2015). Driver gaze tracking and eyes off the road detection system. IEEE Trans. Intell. Transp. Syst..

[29] Naqvi R.A., Arsalan M., Batchuluun G., Yoon H.S., Park K.R. (2018). Deep learning-based gaze detection system for automobile drivers using a NIR camera sensor. Sensors.

[30] Jiménez P., Bergasa L.M., Nuevo J., Hernández N., Daza I.G. (2012). Gaze fixation system for the evaluation of driver distractions induced by IVIS. IEEE Trans. Intell. Transp. Syst..

[31] Hofbauer M., Kuhn C.B., Püttner L., Petrovic G., Steinbach E. Measuring Driver Situation Awareness Using Region-of-Interest Prediction and Eye Tracking. Proceedings of the 2020 IEEE International Symposium on Multimedia (ISM).

[32] Langner T., Seifert D., Fischer B., Goehring D., Ganjineh T., Rojas R. Traffic awareness driver assistance based on stereovision, eye-tracking, and head-up display. Proceedings of the 2016 IEEE International Conference on Robotics and Automation (ICRA).

[33] Mori M., Miyajima C., Angkititrakul P., Hirayama T., Li Y., Kitaoka N., Takeda K. Measuring driver awareness based on correlation between gaze behavior and risks of surrounding vehicles. Proceedings of the 2012 15th International IEEE Conference on Intelligent Transportation Systems.

[34] Quigley M., Conley K., Gerkey B., Faust J., Foote T., Leibs J., Wheeler R., Ng A.Y. ROS: An open-source Robot Operating System. Proceedings of the ICRA Workshop on Open Source Software.

[35] Billings S., Korenberg M., Chen S. (1988). Identification of non-linear output-affine systems using an orthogonal least-squares algorithm. Int. J. Syst. Sci..

[36] Fang J., Yan D., Qiao J., Xue J., Wang H., Li S. DADA-2000: Can Driving Accident be Predicted by Driver Attentionƒ Analyzed by A Benchmark. Proceedings of the 2019 IEEE Intelligent Transportation Systems Conference (ITSC).

[37] Nagaraju D., Ansah A., Ch N.A.N., Mills C., Janssen C.P., Shaer O., Kun A.L. How will drivers take back control in automated vehicles?. A driving simulator test of an interleaving framework. In Proceedings of the 13th International Conference on Automotive User Interfaces and Interactive Vehicular Applications.

[38] Wan J., Wu C. (2018). The Effects of Lead Time of Take-Over Request and Nondriving Tasks on Taking-Over Control of Automated Vehicles. IEEE Trans. Hum. Mach. Syst..

[39] Romera E., Alvarez J.M., Bergasa L.M., Arroyo R. (2017). Erfnet: Efficient residual factorized convnet for real-time semantic segmentation. IEEE Trans. Intell. Transp. Syst..

[40] Chen L., Papandreou G., Schroff F., Adam H. (2017). Rethinking Atrous Convolution for Semantic Image Segmentation. arXiv.

[41] Zabihi S., Zabihi S., Beauchemin S.S., Bauer M.A. Detection and recognition of traffic signs inside the attentional visual field of drivers. Proceedings of the 2017 IEEE Intelligent Vehicles Symposium (IV).

[42] ScenarioRunner Is a Module That Allows Traffic Scenario Definition and Execution for the CARLA Simulator. https://carla-scenariorunner.readthedocs.io/en/latest/.

[43] Blackman S., Popoli R. (1999). Design and Analysis of Modern Tracking Systems.

[44] Hooey B.L., Gore B.F., Wickens C.D., Scott-Nash S., Socash C., Salud E., Foyle D.C. (2011). Modeling pilot situation awareness. Human Modelling in Assisted Transportation.

